# Treatment outcomes and late toxicities of 869 patients with nasopharyngeal carcinoma treated with definitive intensity modulated radiation therapy: new insight into the value of total dose of cisplatin and radiation boost

**DOI:** 10.18632/oncotarget.5420

**Published:** 2015-10-14

**Authors:** Xiaomin Ou, Xin Zhou, Qi Shi, Xing Xing, Youqi Yang, Tingting Xu, Chunying Shen, Xiaoshen Wang, Xiayun He, Lin Kong, Hongmei Ying, Chaosu Hu

**Affiliations:** ^1^ Department of Radiation Oncology, Fudan University Shanghai Cancer Center, Shanghai, China; ^2^ Department of Oncology, Shanghai Medical College, Shanghai, China; ^3^ Department of Radiation Oncology, Shanghai Proton and Heavy Ion Center, Shanghai, China

**Keywords:** nasopharyngeal carcinoma, intensity-modulated radiation therapy, chemotherapy, radiation boost, late toxicity

## Abstract

This study was to report the long-term outcomes and toxicities of nasopharyngeal carcinoma (NPC) treated with intensity-modulated radiation therapy (IMRT). From 2009 to 2010, 869 non-metastatic NPC patients treated with IMRT were retrospectively enrolled. With a median follow-up of 54.3 months, the 5-year estimated local recurrence-free survival (LRFS), regional recurrence-free survival (RRFS), distant metastasis-free survival (DMFS), disease-free survival (DFS) and overall survival (OS) were 89.7%, 94.5%, 85.6%, 76.3%, 84.0%, respectively. In locally advanced NPC, gender, T, N, total dose of cisplatin more than 300 mg/m^2^ and radiation boost were independent prognostic factors for DMFS and DFS. Age, T, N and total dose of cisplatin were independent prognostic factors for OS. Radiation boost was an adverse factor for LRFS, RRFS, DMFS and DFS. Concurrent chemotherapy was not an independent prognostic factor for survival, despite marginally significant for DMFS in univariate analysis. Concurrent chemotherapy increased xerostomia and trismus, while higher total dose of cisplatin increased xerostomia and otologic toxicities. In conclusion, IMRT provided satisfactory long-term outcome for NPC, with acceptable late toxicities. Total dose of cisplatin was a prognostic factor for distant metastasis and overall survival. The role of concurrent chemotherapy and radiation boost in the setting of IMRT warrants further investigation.

## INTRODUCTION

Nasopharyngeal carcinoma (NPC) is an endemic malignancy in Southeast Asia and North Africa. The mainstay of treatment of NPC is radiotherapy. The location of tumor is proximate to multiple critical structures and patients often present with bulky disease, which makes it difficult to achieve satisfactory local control by conventional two dimensional radiotherapy (2DRT). In the era of 2DRT, the 5-year overall survival was 59–69% and local recurrence-free survival (LRFS) was 60.8–79% [1, 2]. Intensity-modulated radiation therapy (IMRT) is a major breakthrough of radiation technique in the past decade. IMRT is able to generate inhomogeneous and conformal radiation beam to fit the area of cancer and spare the adjacent normal tissues with a steep dose gradient. A number of preliminary studies demonstrated improvement of local control by IMRT in NPC [3–6]. The phase II trial of RTOG 0225 demonstrated excellent local control above 90% and 2-year overall survival of 80.4% [6]. Recently, Zhao et al. [7] reported the 5-year disease specific survival of 84.8% and local control of 92.7% in a cohort of 419 NPC patients treated with IMRT. Subsequently, several studies [7–10] reported the 5-year local control and overall survival rates were 86–91.8% and 77.1–84.7% in a large cohort of patients. However, the enrollment of these studies lasted for a few years [7–10] and the techniques of imaging and systemic therapy have evolved during this period. In some studies, head and neck CT was used as the initial staging technique [7, 10] that may influence the staging and evaluation of outcomes.

IMRT has significantly reduced the incidence of xerostomia, compared with 2DRT [11, 12]. However, relative high incidences of late toxicities were reported by some of studies, such as hearing impairment [8, 10] and temporal lobe injury [10, 13]. In order to better understand the efficacy and late toxicities of IMRT, we describe here a consecutive cohort of 869 patients with non-metastatic NPC treated by definitive IMRT in our institution during 2009 to 2010. This was a retrospective analysis of a large size of patients enrolled in a relatively short period of time, with up-to-dated imaging and treatment modalities, aiming to evaluate long-term outcome of IMRT in the current setting and serve as a basis for future study.

## RESULTS

### Treatment outcome

Patient characteristics were illustrated in Table 1. All patients underwent MRI of head and neck at initial diagnosis. 84.8% of patients received cisplatin-based chemotherapy. With a median follow-up of 54.3 months (range, 1.0–75.1 months), the 5-year estimated OS and DFS were 84.0% and 76.3%. The 1, 2, 3, 5-year LRFS were 98.2%, 96.3%, 94.5% and 89.7%. The 1, 2, 3, 5-year RRFS were 99.0%, 97.7%, 96.9% and 94.5%. The 1, 2, 3, 5-year DMFS were 95.2%, 90.7%, 88.9% and 85.6%, respectively.

**Table 1:** Patient and treatment characteristics

Characteristics	Number	Percentage
Patient characteristic		
Age mean (year)	49	
range	12–80	
Gender male	644	74.1%
female	225	25.9%
Pathology		
WHO I	1	0.1%
WHO II-III	868	99.9%
T T1	209	24.1%
T2	317	36.5%
T3	224	25.8%
T4	119	13.7%
N N0	191	22.0%
N1	319	36.7%
N2	250	28.8%
N3	109	12.5%
Stage I	51	5.9%
II	265	30.5%
III	331	38.1%
IVA	113	13.0%
IVB	109	12.5%
Diagnositc imaging technique		
MRI	869	100%
Surgery before radiotherapy		
Nodal excision	46	5.3%
Radiotherapy		
Total prescription dose(Gy) (mean+/−SD)		
primary tumor	68.26+/−3.51	
metastatic lymph nodes	66.37+/−1.37	
Additional boost		
Nasopharyngeal boost	51	5.9%
Nodal boost	72	8.3%
Unplanned break during radiotherapy > 3 days	61	7.0%
Completion of radiation	869	100%
Chemotherapy	737	84.8%
Chemotherapy strategy		
Induction plus concurrent chemotherapy	297	34.2%
Induction plus adjuvant chemotherapy	246	28.3%
Induction plus radiation alone	101	11.6%
Concurrent with/without adjuvant chemotherapy	93	10.7%
Total dose of cisplatin (mg/m^2^) (mean+/−SD)	231.9+/−132.1	

For patients who experienced local, regional or distant failure, the median time to relapse were 31.4 months (range, 3.0–73.2 months), 26.8 months (range, 5.9–60.4 months) and 16.9 months (range, 1.0–66.8 months), respectively. 41.7% of local relapses and 47.4% of regional recurrences and 69.7% of distant metastases occurred within the first two years. Local, regional and distant failures outside the first five years were rare (6.9%, 2.6% and 1.8%, respectively).

The 5-year estimated LRFS, RRFS, DMFS and DFS among various T, N and stage subgroups were listed in table 2. There was no significant difference of LRFS among various T stages (χ^2^ = 3.821, *p* = 0.281). Compared with T1, patients with T4 disease had a marginally higher risk of local relapse (χ^2^ = 3.241, *p* = 0.072). No significant difference was observed between other T subgroups. The RRFS and DMFS were significant different among various N stages (χ^2^ = 19.497, *p* = 0.000; χ^2^ = 27.258, *p* = 0.000). Compared with N1, patients with N2 had a significantly higher rate of regional recurrence (χ^2^ = 6.636, *p* = 0.010) and distant metastasis (χ^2^ = 15.037, *p* = 0.000). Similarly, patients with N3 disease had an obviously higher incidence of regional (χ^2^ = 7.101, *p* = 0.008) and distant failure (χ^2^ = 15.691, *p* = 0.000) compared with the N1 counterparts. There was no significant difference between the N2 and N3 subgroups in terms of regional relapse (χ^2^ = 0.225, *p* = 0.636) and distant metastasis (χ^2^ = 0.472, *p* = 0.492).

**Table 2:** The 5-year estimated survivals among various subgroups

5y LRFS	Rate (%)	5y RRFS	Rate (%)	5y DMFS	Rate (%)	5y DFS	Rate (%)
T1	91.1	N0	99.4	N0	91.5	I	90.9
T2	91.3	N1	96.4	N1	91.0	II	90.2
T3	89.7	N2	91.2	N2	78.6	III	72.5
T4	83.2	N3	86.8	N3	76.2	IVA/IVB	60.5/65.1
Overall	89.7	Overall	94.5	Overall	85.6	Overall	76.3
χ ^2^	3.821	χ ^2^	19.497	χ ^2^	27.258	χ ^2^	46.046
*p* value	0.281	*p* value	0.000	*p* value	0.000	*p* value	0.000

It was noteworthy that radiation was delivered to the primary site and the upper neck (above cricoid cartilage) in N0 patients and the 5-year regional control of this subgroup was excellent (5-year RRFS 99.4%). Only one case developed in-field nodal relapse of the upper neck, synchronously with primary recurrence.

### Failure pattern

During follow-up, there were 116 deaths and 177 treatment failures. The major pattern of failure was isolated distant metastasis (*n* = 91, 51.4%), followed by isolated local recurrence (*n* = 38, 21.5%), local and regional relapse (*n* = 19, 10.7%) and isolated nodal recurrence (*n* = 11, 6.2%), et al. The most frequent sites of metastasis were bone (*n* = 48, 44.0%), lung (*n* = 42, 38.5%), liver (*n* = 43, 39.4%), distant lymph node (*n* = 5, *n* = 4.6%), epidural and spine (*n* = 2, 1.8%). Patterns of failure and sites of distant metastasis were illustrated in Table 3 and 4.

**Table 3:** Failure patterns of all patients

Pattern	Number (percentage, %)
Local recurrence	38 (21.5%)
Local + nodal recurrence	19 (10.7%)
Local + distant relapse	10 (5.6%)
Local, nodal and distant relapse	4 (2.3%)
Nodal recurrence	11 (6.2%)
Nodal recurrence+ distant relapse	4 (2.3%)
Distant relapse	91 (51.4%)

**Table 4:** Sites of distant metastasis (*n* = 109)

Sites of distant metastasis	Number
Solitary	72
Bone	25
Lung	26
Liver	15
Distant lymph nodes	6
Two sites	32
Bone, lung	3
Bone, liver	16
Lung, liver	7
Lung and distant lymph nodes	3
Liver and distant lymph nodes	2
Epidural and spine	2
Multiple sites	5
Bone, lung, liver	3
Others	1

### Chemotherapy

95.3% of patients with locally advanced NPC received chemotherapy. The strategies and regimens of chemotherapy were listed in Table 5. The most common strategies in our institution were induction chemotherapy plus concurrent chemoradiation (CCRT) (44.1%) and induction chemotherapy plus adjuvant chemotherapy (34.4%), followed by induction chemotherapy and radiation (10.7%) as well as CCRT with/without adjuvant chemotherapy (6.1%). During induction chemotherapy, 60.5% of patients were treated with docetaxel-comprising chemotherapy, while 13.4% received gemcitabine-comprising regimen. During adjuvant chemotherapy, 43.9% of patients were administrated with docetaxel-comprising regimen, while 28.8% were treated with gemcitabine-comprising chemotherapy.

**Table 5:** Strategies and regimens of chemotherapy in locally advanced nasopharyngeal carcinoma

Characteristics	Number	Percentage
Strategies		
Induction CT. + RT.	59	10.7%
Induction CT. + CCRT.	244	44.1%
Induction CT. + Adjuvant CT.	190	34.4%
CCRT. +/–Adjuvant CT.	34	6.1%
RT. alone†	26	4.7%
Regimens for induction CT.		
TPF/TP	335	60.5%
GP	74	13.4%
PF	79	14.3%
Others‡	5	0.9%
Regimens for adjuvant CT.		
TPF/TP	90	43.9%
GP	59	28.8%
PF	56	27.3%

† Only patients with severe comorbidities or elderly patients that could not tolerate chemotherapy received radiation alone.

‡ The regimen of induction chemotherapy was changed due to adverse effects in three patients. Two patients received induction chemotherapy in other hospital and the concrete regimen was not specified in medical history.

Abbreviation: CT. = chemotherapy; CCRT. = concurrent chemotherapy; RT. = radiation

There was no significant difference of local or regional control among various regimens of induction chemotherapy (Table 6). The 5-year DMFS of TPF/TP, GP and PF during induction chemotherapy were 81.4%, 82.3% and 72.2% (*p* = 0.266), respectively. The 5-year OS showed a trend of improving survival in the subgroup of TPF/TP and GP, although this did not reach a significant level (TPF/TP vs. GP vs. PF: 84.1% vs. 80.0% vs. 72.2%, *p* = 0.133, supplementary Figure S1A). In addition, the 5-year OS was marginally higher in the category of induction chemotherapy comprising docetaxel or gemcitabine (83.3% vs. 72.2%, *p* = 0.058, supplementary Figure S1B). There was no significant difference of survival rates among various regimens of adjuvant chemotherapy.

**Table 6:** The 5-year estimated survivals stratified by various regimens of chemotherapy of locally advanced nasopharyngeal carcinoma

5y rates	LRFS	RRFS	DMFS	OS
Induction chemotherapy				
TPF/TP	88.7%	92.8%	81.4%	84.1%
GP	82.7%	93.0%	82.3%	80.0%
PF	85.8%	93.6%	72.2%	72.2%
χ^2^	0.331	0.044	2.645	4.038
*p* value	0.848	0.978	0.266	0.133
Adjuvant chemotherapy				
TPF/TP	88.3%	90.1%	73.7%	86.6%
GP	84.9%	90.9%	82.5%	80.9%
PF	87.4%	95.3%	81.4%	81.8%
χ^2^	0.024	0.388	0.240	0.813
*p* value	0.988	1.895	2.852	0.413

### Prognostic analysis

Our study demonstrated excellent result in stage I-II patients, with a 5-year DFS above 90%. However, the long-term outcomes of stage III-IVB cases were much poorer, with a 5-year DFS about 60–70%. In order to figure out prognostic factors of locally advanced NPC, univariate and multivariate analyses were conducted (Table 7, 8).

**Table 7:** Univariate analysis of various clinical factors on survivals of locally advanced nasopharyngeal carcinoma (*n* = 553)

	Number	5y LRFS	*p* value	5y RRFS	*p* value	5y DMFS	*p* value	5y DFS	*p* value	5y OS	*p* value
Age											
≤ 48	274	84.6%	0.060	92.7%	0.913	77.9%	0.236	67.1%	0.221	83.1%	0.040*
> 48	279	90.1%		92.0%		81.3%		69.9%		76.4%	
Gender											
male	409	85.5%	0.039*	92.6%	0.855	76.1%	0.006*	65.2%	0.005*	77.5%	0.152
female	144	93.1%		91.6%		89.5%		78.0%		86.4%	
T category											
T1–3	434	88.7%	0.266	91.3%	0.083	82.0%	0.030*	71.0%	0.071	80.8%	0.591
T4	119	83.2%		96.4%		70.7%		59.7%		76.5%	
N category											
N0–1	194	85.7%	0.631	96.6%	0.015*	82.9%	0.149	71.8%	0.164	83.7%	0.212
N2–3	359	88.4%		90.1%		77.9%		66.8%		77.7%	
Stage											
III	331	88.0%	0.531	92.6%	0.155	83.5%	0.011*	72.5%	0.032*	82.2%	0.174
IV	222	86.6%		87.0%		73.3%		62.3%		75.9%	
Chemotherapy											
yes	527	87.0%	0.053	96.2%	0.711	80.0%	0.044*	68.4%	0.568	80.7%	0.002*
no	26	100%		92.2%		69.8%		69.8%		60.5%	
Concurrent chemotherapy											
yes	278	87.3%	0.258	92.7%	0.823	83.6%	0.050	71.6%	0.266	82.6%	0.082
no	275	87.4%		92.1%		75.7%		65.4%		77.0%	
Induction chemotherapy											
yes	493	87.5%	0.855	93.0%	0.160	79.9%	0.207	69.0%	0.466	80.7%	0.057
no	60	86.4%		87.8%		76.1%		63.9%		71.4%	
Adjuvant chemotherapy											
yes	202	87.6%	0.569	92.2%	0.865	78.0%	0.846	65.4%	0.764	83.5%	0.208
no	351	87.5%		92.5%		80.5%		70.3%		77.4%	
Chemotherapy Strategy											
Induction CT. + RT.	59	83.4%	0.509	87.8%	0.137	73.9%	0.395	63.6%	0.624	62.6%	0.030*
Induction CT. + CCRT.	244	88.8%		93.6%		84.0%		73.5%		83.0%	
Induction CT. + Adjuvant CT.	190	86.9%		93.6%		76.8%		65.3%		83.1%	
CCRT. +/–Adjuvant CT.	34	78.0%		82.8%		81.2%		60.2%		80.0%	
Induction chemotherapy regimens											
TPF/TP/GP	409	87.7%	0.686	92.8%	0.999	81.6%	0.140	69.8%	0.539	83.3%	0.058
PF	79	85.8%		93.6%		72.2%		65.8%		72.2%	
Adjuvant chemotherapy regimens											
TPF/TP/GP	149	88.2%	0.944	92.2%	0.774	77.0%	0.469	66.0%	0.325	84.7%	0.577
PF	56	84.9%		90.9%		82.5%		65.3%		80.9%	
Total dose of cisplatin											
≥ 300 mg/m2	338	86.4%	0.377	93.2%	0.506	82.0%	0.016*	70.0%	0.218	84.2%	0.005*
< 300 mg/m2	215	89.3%		90.8%		75.5%		66.0%		72.3%	
Radiation boost											
yes	74	73.7%	0.004*	83.1%	0.000*	71.2%	0.085	52.2%	0.004*	80.2%	0.995
no	479	89.5%		93.8%		80.9%		71.1%		79.5%	
Radiation interruption > 3 days											
yes	46	92.8%	0.609	92.7%	0.777	82.7%	0.675	73.3%	0.780	69.4%	0.592
no	507	87.0%		92.4%		79.3%		68.1%		80.4%	

Abbreviation: CT. = chemotherapy; CCRT. = concurrent chemotherapy; RT. = radiation.

* indicated *p* < 0.05.

**Table 8:** Multivariate analysis of various clinical factors on survivals of locally advanced nasopharyngeal carcinoma (*n* = 553)

Survival	Factor	*p* value	HR(95%CI)
LRFS	Gender (female vs. male)	0.041	0.451(0.210–0.968)
	Radiation Boost (yes vs. no)	0.004	2.400(1.317–4.376)
RRFS	N stage (N3/N2/N1/N0)	0.034	1.613(1.037–2.508)
	Radiation Boost (yes vs. no)	0.001	3.432(1.653–7.125)
DMFS	Gender (female vs. male)	0.004	0.374(0.191–0.731)
	T stage (T4/T3/T2/T1)	0.002	1.526(1.174–1.984)
	N stage (N3/N2/N1/N0)	0.001	1.616(1.209–2.159)
	Total dose of cisplatin (≥300 vs. < 300 mg/m^2^)	0.004	0.501(0.313–0.800)
	Radiation Boost (yes vs. no)	0.009	2.085(1.200–3.622)
DFS	Gender (female vs. male)	0.004	0.515(0.327–0.811)
	T stage (T4/T3/T2/T1)	0.016	1.650(1.096–2.485)
	N stage (N3/N2/N1/N0)	0.028	1.567(1.049–2.342)
	Total dose of cisplatin (≥300 vs. < 300 mg/m^2^)	0.036	0.688(0.486–0.976)
	Radiation boost (yes vs. no)	0.001	1.932(1.290–2.892)
OS	Age (>48 vs. ≤ 48)	0.031	1.662(1.047–2.639)
	T stage (T4/T3/T2/T1)	0.024	1.361(1.041–1.779)
	N stage (N3/N2/N1/N0)	0.001	1.699(1.254–2.300)
	Total dose of cisplatin (≥300 vs. < 300 mg/m^2^)	0.012	0.554(0.350–0.879)

**Figure 1:**
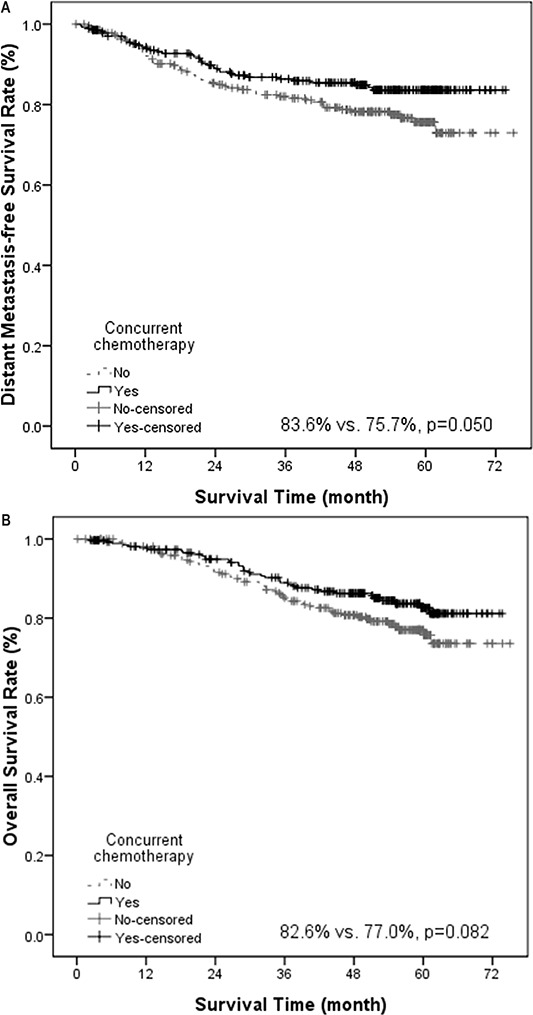
Kaplan-Meier estimate of distant metastasis–free survival (A) and overall survival (B) stratified by concurrent chemotherapy

**Figure 2:**
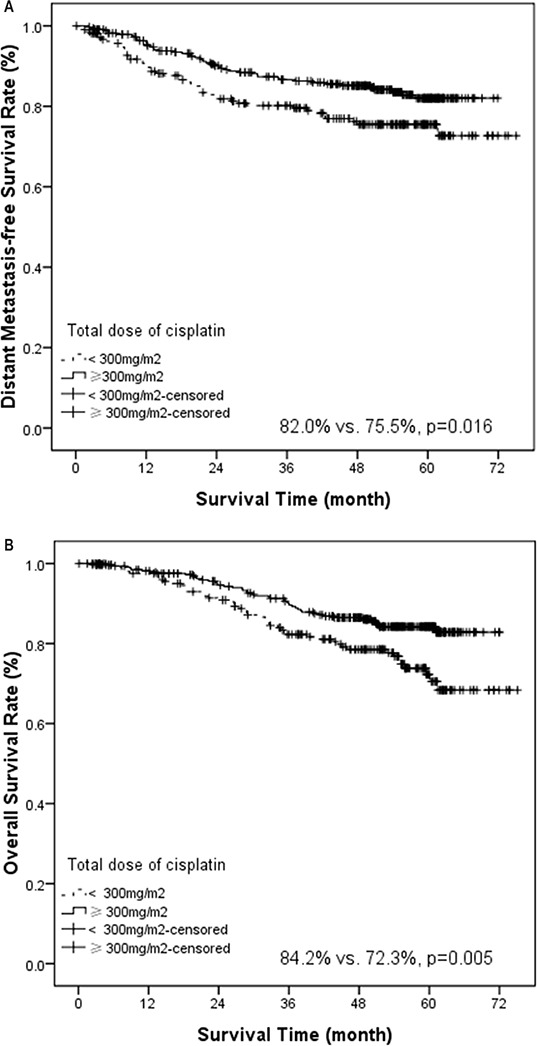
Kaplan-Meier estimate of distant metastasis-free survival (A) and overall survival (B) stratified by total dose of cisplatin (total dose ≥ 300 mg/m^2^ vs. < 300 mg/m^2^)

**Figure 3:**
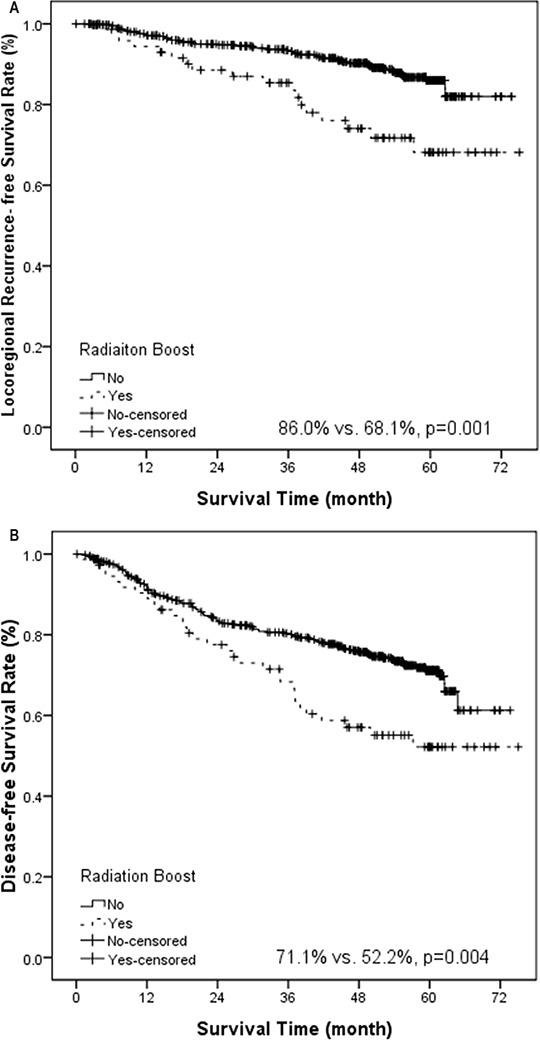
Kaplan-Meier estimate of locoregional recurrence-free survival (A) and disease-free survival (B) stratified by radiation boost

Univariate analysis revealed that concurrent chemotherapy marginally improved DMFS (83.6% vs. 75.7%, *p* = 0.050, Figure 1A) and had a trend of improving OS (82.6% vs. 77.0%, *p* = 0.082, Figure 1B). Induction chemotherapy marginally improved OS (80.7% vs. 71.4%, *p* = 0.057). Adjuvant chemotherapy did not confer significant impact on various survival rates. If we analyzed the survival rates based on combination of chemotherapy, the 5-year OS was significantly different among various treatment strategies (*p* = 0.030, Supplementary Figure S2). In addition, patients treated with more than 300 mg/m^2^ of cisplatin had a better distant control (82.0% vs. 75.5%, *p* = 0.016, Figure 2A) and overall survival (84.2% vs. 72.3%, *p* = 0.005, Figure 2B). Radiation boost was associated with a lower LRFS (73.7% vs. 89.5%, *p* = 0.004), RRFS (83.1% vs. 93.8%, *p* = 0.000) and DFS (52.2% vs. 71.1%, *p* = 0.004, Figure 3).

Multivariate analysis showed that gender, T stage, N stage, total dose of cisplatin and radiation boost were independent prognostic factors for both DMFS and DFS. Age, T stage, N stage and total dose of cisplatin were independent prognostic factors for OS. The predictive value of concurrent chemotherapy and treatment strategy was not statistically significant in multivariate analysis. However, total dose of cisplatin was an independent prognostic factor for DMFS, DFS and OS. Radiation boost had an adverse impact on LRFS, RRFS, DMFS and DFS.

In order to rule out the impact of dosimetric difference between the patients with or without radiation boost, dosimetric analysis was performed for the PTVs and V95 (volume that receives more than 95% of prescription dose) (Supplementary Table S1). There was no significant difference of PTVs and V95 between the two groups.

### Major late toxicities

Table 9 summarized the 5-year rates of major late toxicities. 810 patients with records of toxicity evaluation and more than one year of follow-up were included into analysis. The incidence of any grade of xerostomia, hearing impairment, cranial nerve injury, temporal lobe necrosis was 40.7%, 21.6%, 2.7% and 5.6%, respectively. Concurrent chemotherapy significantly increased xerostomia (any grade: 46.4% vs. 36.3%, *p* = 0.003; grade 3 to 4: 5.6% vs. 2.7%, *p* = 0.034) and trismus (any grade: 2.8% vs. 0.2%, *p* = 0.001). The total dose of cisplatin had a significant impact on late toxicities. Higher total dose increased xerostomia (any grade: 44.1% vs. 37.5%, *p* = 0.035) and hearing impairment (any grade: 25.1% vs. 18.2%, *p* = 0.013; grade 3 to 4: 4.8% vs. 1.9%, *p* = 0.026).

**Table 9:** Frequency of late toxicities (CTCAE 3.0) in patients with more than one year of follow-up (*n* = 810)

Toxicity	Any Grade	Grade 3–4	Any Grade: CCRT vs. Non-CCRT	*p* value	Grade 3–4: CCRT vs. Non-CCRT	*p* value	Any Grade: Total dose of cisplatin ≥ 300 mg/m^2^vs. < 300 mg/m^2^	*p* value	Grade 3–4: Total dose of cisplatin ≥ 300 mg/m^2^vs. < 300 mg/m^2^	*p* value
Xerostomia	330 (40.7%)	32 (4.0%)	46.4% vs. 36.3%	0.003*	5.6% vs. 2.7%	0.034*	44.1% vs. 37.5%	0.035*	5.0% vs. 2.9%	0.127
Hearing impairment	175 (21.6%)	27 (3.3%)	24.0% vs. 19.7%	0.132	3.9% vs. 2.9%	0.659	25.1% vs. 18.2%	0.013*	4.8% vs. 1.9%	0.026*
Hearing loss	105 (13.0%)	14 (1.7%)	14.8% vs. 11.5%	0.170	2.0% vs. 1.5%	0.261	15.5% vs. 10.5%	0.026*	2.8% vs. 0.7%	0.027*
Tinnitus	77 (9.5%)	1 (0.1%)	10.1% vs. 8.8%	0.467	0.3% vs. 0%	0.788	11.5% vs. 7.5%	0.052	0.3% vs. 0%	0.493
Otitis media	26 (3.2%)	14 (1.7%)	4.5% vs. 2.2%	0.074	2.0% vs. 1.5%	0.659	3.3% vs.3.2%	0.929	2.0% vs. 1.5%	0.599
Cranial nerve injury	22 (2.7%)	19 (2.3%)	3.6% vs. 2.2%	0.152	3.1% vs. 1.8%	0.224	3.8% vs. 1.7%	0.073	3.0% vs. 1.7%	0.220
Temporal lobe necrosis	45 (5.6%)	7 (0.9%)	6.1% vs. 5.1%	0.519	0.8% vs. 0.8%	0.943	6.0% vs. 5.1%	0.562	1.0% vs. 0.7%	0.722
Trismus	11 (1.4%)	2 (0.2%)	2.8% vs. 0.2%	0.001*	0.6% vs. 0%	0.195	1.8% vs. 1.0%	0.299	0.5% vs. 0%	0.242
Secondary malignancy	6 (0.7%)	6 (0.7%)	1.4% vs. 0.2%	0.093	1.4% vs. 0.2%	0.093	1.3% vs. 0.2%	0.118	1.2% vs. 0.2%	0.118

Abbreviation: CCRT. = concurrent chemotherapy.

* indicated *p* < 0.05.

There were 45 cases (crude incidence, 5.6%) of MRI-evidenced temporal lobe necrosis during follow-up. Of them, 36 cases were asymptomatic, 9 cases complaint of headache and memory decline, one case complaint of personality change and dementia. The median latency of temporal lobe necrosis was 39.3 months (range, 3.5–71.9 months). Univariate analysis showed that T stage was a prognostic factor of temporal lobe necrosis. The 5-year temporal lobe necrosis-free survival rates for T1, T2, T3, T4 were 98.8%, 92.6%, 90.4% and 84.7% (χ^2^ = 12.429, *p* = 0.006), respectively. Concurrent chemotherapy and radiation boost did not confer significant increase on temporal lobe necrosis.

## DISCUSSION

### Long-term outcomes of IMRT

The application of IMRT in NPC was first reported by University of California-San Francisco in 2000, which showed encouraging result of 100% locoregional control and 4-year OS of 94% [3]. Subsequently, a series of preliminary studies reported 3 to 4 year local control above 90% and OS of 80–90% [4, 14]. A phase III randomized trial [15] comparing IMRT and 2DRT showed that the 5-year LRFS was increased from 83.8% to 90.5% and OS was increased from 67.1% to 79.6% by IMRT. Similarly, Lee et al. [8] performed a retrospective analysis of 1593 patients and found significant improvement in local control by IMRT. About 10% of improvement of LRFS was observed among all T categories.

In accordance with previous studies, our study demonstrated a 5-year local control of 89.7% and 5-year OS of 84.0%. The locoregional control of T1-T3 achieved about 90% and that of T4 was 83.0%. No significant difference of LRFS was observed among various T stages, despite marginal difference of T1 and T4. Similarly, Lin et al. [16] reported that T-classification was no longer a significant prognostic factor for local control in the setting of IMRT. This was probably attributed to the technical advantage of IMRT. IMRT is able to deliver higher and more conformal dose to tumor volume that translated into better local control. In addition, the simultaneous integrated boost IMRT used in this study increased fractionated dose and shortened treatment course that might translate into locoregional gain. However, in T4 cases, the dose coverage of intracranial tumor volume is limited by nearby critical normal structure. In addition, higher percentage of hypoxic cells in larger tumor influences local control. Further research is warranted to improve the local control of T4 disease. Neoadjuvant chemotherapy and replanning IMRT is probably a feasible strategy for intracranial invasion [17].

### The role of chemotherapy in the era of IMRT

In the era of 2DRT, the main failure patterns were local recurrence and distant metastasis [18]. In the era of IMRT, the major failure pattern shifted to be distant metastasis [10, 16]. Our study demonstrated that N stage was an independent prognostic factor for DMFS, as was reported in previous studies [10, 16]. The N2–3 category had a higher risk of distant metastasis. Thus, more aggressive systemic treatment is needed to achieve distant control in this group of patients. In our study, total dose of cisplatin more than 300 mg/m^2^ in the whole course of treatment was found to be an independent prognostic factor for DMFS, DFS and OS. To our knowledge, this is the first report of adequate cisplatin dose to achieve disease control in the setting of IMRT. In head and neck cancer, a necessary dose threshold of 200 mg/m^2^ of cisplatin has been suggested to achieve satisfactory disease control [19]. However, the adequate dose of cisplatin has not been clarified in NPC. The combined analysis [20] of NPC-9901 [21] and NPC-9902 [22] trials revealed that the dose of concurrent cisplatin 200 mg/m^2^ was a significant factor for locoregional failure and overall survival. Given to the higher proportion of distant metastasis in NPC, compared with other head and neck cancer, it is reasonable to suggest total dose of cisplatin 300 mg/m^2^ might be required to achieve satisfactory distant control and overall survival.

Since the publication of the landmark Intergroup 0099 trial [23], the value of concurrent chemotherapy has been repeatedly proven by various clinical studies [21, 24–27]. The Meta-Analysis of Chemotherapy in Nasopharynx Carcinoma (MAC-NPC) [28] showed that the combination of chemotherapy has brought an absolute survival benefit of 6% after 5 years, whereas the most effective approach was concurrent chemotherapy. Recently, an update of MAC-NPC [29] confirmed that concomitant chemotherapy significantly improves progression-free survival and overall survival. However, most clinical trials [22, 25, 27] were performed in the setting of 2DRT. More than three quarters of trials in the updated study of MAC-NPC [29] were based on 2DRT. Thus, the benefit of concurrent chemotherapy in the era of IMRT remains controversial. Lin [30] et al. reported that concurrent chemotherapy provided no significant benefit to IMRT after induction chemotherapy in locoregionally advanced NPC. Recently, the large-scale clinical report [10] of long-term outcomes of IMRT demonstrated no survival benefit of concurrent chemotherapy as well.

In accordance with previous reports, our study demonstrated that concurrent chemotherapy was not an independent prognostic factor for survivals, despite marginally significant for distant control and overall survival in univariate analysis. Detailed analysis showed that induction chemotherapy followed by CCRT, induction plus adjuvant chemotherapy and concurrent chemotherapy with/without adjuvant chemotherapy had similar overall survival, whereas induction chemotherapy and radiation alone had a lower overall survival. Neither did concurrent chemotherapy nor the choice of chemotherapy strategy significantly influence overall survival in multivariate analysis. However, the total dose of cisplatin was essential. The reason for poorer survival of induction chemotherapy and radiation alone was probably inadequate dose of cisplatin. Given the optimal local control and the change of failure patterns by IMRT, the adequate dose of chemotherapy, rather than the timing of chemotherapy, may be more important for distant control and overall survival. Prospective trials to compare these strategies are warranted to confirm this assumption.

New chemotherapy regimen comprising taxane [31, 32] or gemcitabine [33] generated exciting results in NPC. Hui [31] et al. reported that concurrent chemotherapy with taxane-based induction chemotherapy greatly improved overall survival. In head and neck cancer, the TAX 324 trial [34] demonstrated a significantly longer survival in the induction chemotherapy group of TPF, compared with the PF group. In the present study, the 5-year OS of the induction TPF/TP and PF regimen were 84.1% and 72.2%. A trend of better survival in favor of taxane-including regimens was observed. Similarly, the 5-year of OS of the induction GP regimen was 80.0%, displaying a trend of better survival as well. Currently, there is no result of head to head comparison of these regimens of induction chemotherapy in locally advanced NPC. Our results suggest a better survival trend of taxane and gemcitabine-comprising regimen, which is valuable for clinical reference.

### The role of radiation boost in the setting of IMRT

Retrospective analysis showed that additional intracavitary brachytherapy and parapharyngeal boost improved local control in 2DRT [35, 36]. However, the high incidence of late toxicity suggested an overtreatment [37]. In our institution, notable persistent disease at the end of radiation was generally treated with boost irradiation. However, patients with additional boost irradiation were associated with poorer outcomes. The conflicting results were partially attributed to the different dosimetric characteristics of 2DRT and IMRT. The conformal and homogeneous dose distribution of IMRT [38] that brought improvement for local control may counterpart the benefit of radiation boost. It remains questionable whether radiation boost provides additional improvement in the settings of IMRT. To our knowledge, only one retrospective single-armed study [39] demonstrated satisfactory local control by fractionated stereotactic radiotherapy after primary IMRT. Another explanation was the intensive systemic treatment currently used. Induction chemotherapy has greatly shrunken tumor volume [31] so that a lower dose of radiation may be enough to achieve local control. In addition, the residual tumor after intensive chemoradiation probably suggested a more treatment-reluctant phenotype. This was consistent with the study of Zhang [40] et al., which demonstrated that patients with persistent disease at the end of radiation had a significantly poorer disease control and overall survival. Boost irradiation did not confer any survival benefit to patients with persistent disease. Therefore, the benefit of radiation boost in IMRT should be re-evaluated. Perhaps more individualized strategy should be developed for patients with residual disease. Selective adjuvant chemotherapy in the subgroup with persistent EBV DNA load might be a better treatment strategy [41] and requires further investigation.

### Late toxicities of IMRT

Numerous studies have shown that IMRT exhibited advantages for reducing most late toxicities [8, 11, 15]. Our study showed the incidences of xerostomia, trismus, hearing impairment, temporal lobe necrosis and cranial nerve neuropathy were comparable to earlier reports of IMRT [8, 10, 13, 15]. However, recent studies revealed that concurrent chemotherapy increased the grade and incidence of late toxicities including xerostomia [13], hearing impairment [10, 13], trismus [13] and temporal lobe necrosis [13]. In line with this, our study demonstrated that concurrent chemotherapy increased trismus and ≥ grade 3 of xerostomia. Higher total dose of cisplatin increased the rates of late otologic toxicities. An increase of any grade and ≥ grade 3 of hearing impairment was found in the higher cisplatin subgroup. Similarly, Lee [8] et al. demonstrated that concurrent chemotherapy was strong predictive factor for deafness and higher proportion of concurrent chemotherapy resulted into remarkable increase of ≥ grade 3 hearing loss. Therefore, the significant increase of hearing impairment by large dose of chemotherapy should be noticed. Perhaps a more stringent dose-volume constraint is needed when combing with chemotherapy.

In the present study, the incidence of temporal lobe necrosis was 5.6% (crude, 5-year estimated incidence of 7.3%), which was comparable to 5.4%-13.1% in the previous IMRT reports [10, 13, 15]. Compared to our previous report of 2DRT (4-year actuarial incidence was 15.3%; data not published), these results appeared quite acceptable as well. The relatively low incidence of temporal lobe necrosis was probably attributed to small volume of CTV, which was similar to reduced-volume IMRT reported by Lin et al. [16], as well as the stringent dose constraint of temporal lobe in our institution. In addition, T stage was shown to be a prognostic factor of temporal lobe necrosis in our study and patients with advanced T stage were had a higher incidence of temporal lobe necrosis. Similarly, Sun [10] et al. and Basket [42] et al. reported radiation-induced temporal lobe encephalopathy was most likely to happen in patients with advanced T-stage disease. Further analysis by Basket [42] et al. revealed that the location of temporal lobe necrosis was just within the areas of prescription dose or adjacent to PTV. Thus, it is possible that the close margin between primary tumor and brain tissue, makes it difficult to achieve high dose in tumor volume meanwhile spare the infield or marginal in-filed brain tissue. What's more, our recent study demonstrated that V45 (volume that receives more than 45Gy) was also a strong predictive factor of temporal lobe necrosis [43]. Thus, the larger area of moderate dose coverage of brain tissue in advanced T cases partially accounted for this phenomenon as well.

### Conclusion

Taken together, our study demonstrated that IMRT provided satisfactory local control for NPC, with acceptable late toxicities. The main failure pattern was distant metastasis. Concurrent chemotherapy was not an independent prognostic factor for survival, despite marginally significant for reducing distant metastasis. The total dose of cisplatin was an independent prognostic factor for distant metastasis and overall survival. Patients with persistent disease after primary radiation and treated with radiation boost had a higher risk of disease relapse. The role of radiation boost in the settings of IMRT warrants further investigation. The incidences of late toxicities were reduced by IMRT, compared with historical reports of 2DRT. However, the frequencies of late toxicities, especially otologic impairment were increased by administration of chemotherapy.

## MATERIALS AND METHODS

### Patients and pretreatment evaluations

From January 2009 through December 2010, a total of 869 patients that fulfilled the following criteria were consecutively enrolled in the study. Inclusion criteria were pathologically confirmed NPC, previously untreated, no evidence of distant metastasis, no previous malignancy or other concomitant malignant disease, no treatment of molecular targeted therapy, receiving whole course of radical IMRT in our institution.

Routine workup comprised complete medical history, physical examination, indirect or fiberoptic endoscopic examination, complete blood counts, liver and renal functions. MRI scans of head and neck were performed to evaluate the extent of the locoregional disease. All the patients were staged using the 6th edition of the staging system of the American Joint Committee of Cancer (AJCC). Chest CT, abdominal sonography and bone scintigraphy were performed to exclude distant metastasis.

### Radiotherapy techniques

Patients were immobilized in the supine position with a thermoplastic mask. CT was performed after immobilization, obtaining 3-mm slices from the anterior clinoid process to the hyoid bone, and 5-mm slices from the hyoid bone to 2 cm below the sternoclavicular joint. According to the definitions of the ICRU50 and 62 (International Commission on Radiation Units and Measurements), the target volumes were outlined on each layer of the CT images on an IMRT workstation. The gross tumor volume (GTV) included primary tumor and metastatic lymph nodes. The high-risk clinical target volume (CTV) should cover the entire nasopharynx, parapharyngeal space, clivus, base of skull, pterygoid fossa, posterior half of ethmoidal sinus, inferior sphenoid sinus, and posterior edge of nasal cavity and maxillary sinuses. If the tumor involved the inferior sphenoid sinus, the whole sphenoid sinus should be encompassed. The high-risk CTV should include bilateral coverage of levels II, III, VA and RLNs in N0 patients. For patients with metastatic cervical nodes of the upper neck (above cricoid cartilage), the low risk CTV should cover level IV and VB. For individuals with metastatic cervical nodes involving the lower neck (below cricoid cartilage), all the neck levels from II to V were defined as high-risk CTV. A margin of 3–5 mm around GTV and CTV should be added to account for the patient motion and set-up error. A smaller margin will be used for the primary tumor where it is adjacent to a critical neurologic structure. Radiation was delivered using a simultaneous integrated boost-IMRT technique. The total dose to primary tumor was 66 Gy in 30 fractions for T1 or T2 disease, and 70.4 Gy in 32 fractions for T3 or T4 lesion. The total dose to metastatic lymph node was 66Gy in 30–32 fractions. A total dose of 60 Gy and 54 Gy was delivered to the high-risk and low-risk CTV in 30–32 fractions, respectively. Adjacent critical organs, including the brain stem, spinal cord, temporal lobe, optic nerves, optic chiasm, lens, parotid glands, mandible, and temporomandibular joints, were also delineated. Inverse IMRT plans were optimized using Pinnacle (Pinnacle 3; Philips Corp, Fitchburg, WI) treatment planning system. The normal tissue constraints and plan evaluation were in accordance with the Radiation Therapy Oncology Group 0225 protocol [6].

### Management of residual disease

During radiotherapy, physical examination and indirect nasopharyngoscopy were performed every week. At the end of radiotherapy, MRIs of head and neck were conducted. Based on clinical and radiologic examination, nasopharyngeal and neck nodal residual diseases were determined. Local residual diseases were treated by either small-field IMRT or intracavitary afterloading treatment. Small-field IMRT was applied to treat local residual disease just after the planned treatment with 2.2–4.4Gy in one or two daily fractions. Intracavitary afterloading treatment with iridium-192 was used to address local persistence at 2 to 3 weeks after external radiation with 8 to 16 Gy by one or two weekly fractions. Palpable residual nodes present after external radiaiton were treated with a boost of 4–6 Gy in 2 or 3 daily fractions using an electron field of 9 to 12 MeV just after the planned treatment.

### Chemotherapy

All stage I-IIA patients were treated with radiation alone. Cisplatin-based chemotherapy was recommended to medically fit patients with stage IIB–IVB disease. In general, chemotherapy was administered to 84.8% of patients. The details of chemotherapy strategy was illustrated in Table 1. At that time, our institution was conducting a clinical trial comparing the efficacy and toxicities of induction chemotherapy and CCRT with induction chemotherapy and adjuvant chemotherapy. Thus, these two modalities accounted for most.

The regimens of induction and adjuvant chemotherapy included TPF, TP, PF and GP. The TPF protocol consisted of docetaxel 75 mg/m^2^ IV on day 1, cisplatin75 mg/m^2^ IV on day 1, and 5-fu 500 mg/m^2^ d continuously IV on day1–5. The TP protocol consisted of docetaxel 75 mg/m^2^ IV on day 1, cisplatin75 mg/m^2^ IV on day 1. The PF protocol comprised cisplatin 75 mg/m^2^ IV on day 1 and 5-fu 500 mg/m^2^ d continuously IV on day 1–5. The GP regimen included cisplatin 75 mg/m^2^ IV on day 1 and gemcitabine 1000 mg/m^2^ IV on day 1, 8. The regimens were repeated every 3 weeks for 2–3 cycles for induction chemotherapy and every 4 weeks for 2–3 cycles for adjuvant phase. Concurrent chemotherapy consisted of cisplatin 40 mg/m^2^ IV weekly or cisplatin 80 mg/m^2^ every 3 weeks during radiation.

### Follow-up

Patients were followed up after completion of radiotherapy every 3 months in the first 2 years, then every 6 months from year 2 through year 5, and then annually. In each visit, complaint inquery, physical examination including direct or indirect nasopharyngoscopy were performed. MRI of head and neck were required every 3 to 6 months in the first 3 years. The following tests were done at least every year: chest CT, abdominal sonography, bone scan when clinical indicated. Late radiation toxicities were scored according to Common Terminology Criteria for Adverse Events 3.0 version grading system (CTCAE 3.0).

### Statistical analysis

All analyses were performed by SPSS software, version 16.0. The following endpoints were assessed: overall survival (OS), local recurrence-free survival (LRFS), regional recurrence-free survival (RRFS), distant metastasis-free survival (DMFS) and disease-free survival (DFS). All the endpoints were defined as the interval from the date of initiation of treatment to the date of the failure or last follow-up. The Kaplan-Meier method was used to estimate survival rates and the Log rank test was applied to compare the difference. Hazard ratio (HR) and the associated 95% confidence interval (CI) were calculated using Cox proportional hazard model. The χ^2^ test was used for comparing categorical variables, and independent *t*-test was used for comparing the means of continuous variables. In all cases, a 2-sided *p* value < 0.05 was considered statistically significant.

## SUPPLEMENTARY FIGURES AND TABLE


