# Supplementary material for: Treatment outcomes and late toxicities of 869 patients with nasopharyngeal carcinoma treated with definitive intensity modulated radiation therapy: new insight into the value of total dose of cisplatin and radiation boost
Source: Oncotarget. 2015 Oct 14;6(35):38381–97. doi: 10.18632/oncotarget.5420 (PMC4742007; doi:10.18632/oncotarget.5420)
Supplement: Supplementary file 1 [file oncotarget-06-38381-s001.pdf]

SUPPLEMENTARY FIGURES AND TABLE

A

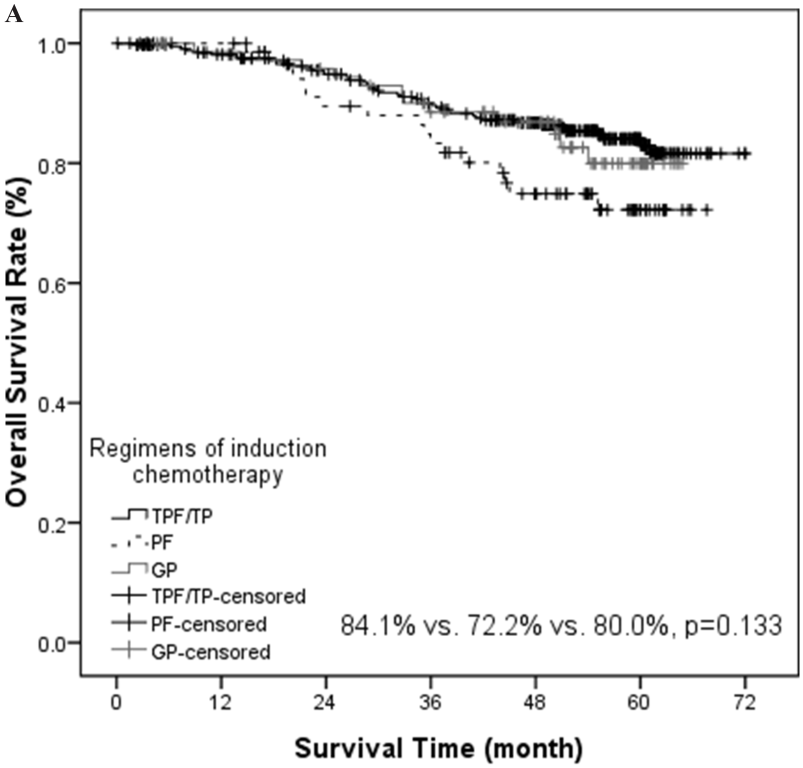

B

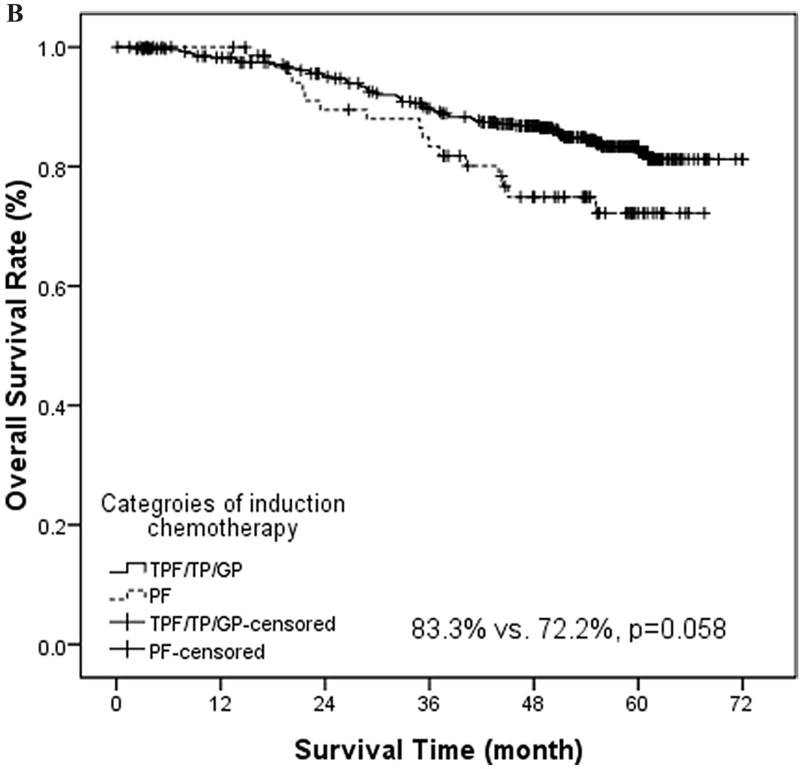

Supplementary Figure S1: Kaplan-Meier estimate of overall survival stratified by various regimens of induction chemotherapy (A) and categories of induction chemotherapy (B).

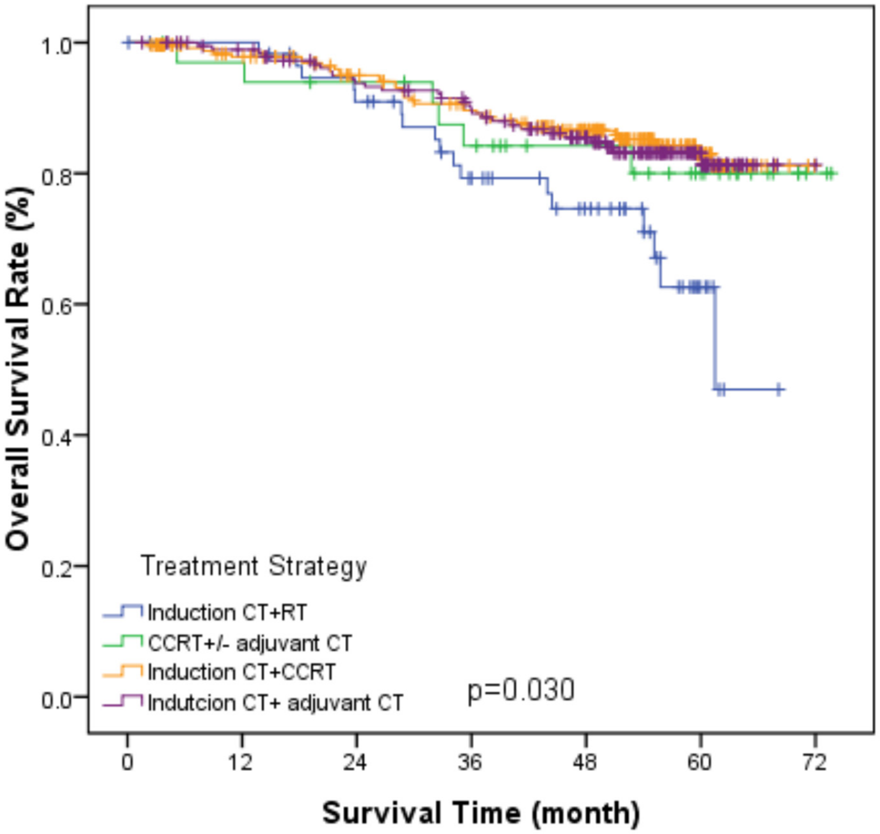

Supplementary Figure S2: Kaplan-Meier estimate of overall survival stratified by treatment strategy. The 5-year overall survival rates of induction chemotherapy plus radiation, induction chemotherapy plus concurrent chemoradiation, induction chemotherapy plus adjuvant chemotherapy and concurrent chemoradiation with/without adjuvant chemotherapy were 62.6%, 83.0%, 83.1% and 80.0%, respectively (p = 0.030). Abbreviation: CT. = chemotherapy; RT. = radiation; CCRT. = concurrent chemoradiation.

Supplementary Table S1: The comparison of dosimetric statistics of the subgroups with or without radiation boost (mean value)

Dosimetric statistics (Gy)	Without boost	With radiation boost*	*p* value
PTV–T_{max}	75.1	74.1	0.167
PTV–T_{min}	60.1	60.9	0.539
PTV–T_{mean}	70.6	70.3	0.592
PTV–LN_{max}	72.7	72.9	0.665
PTV–LN_{min}	55.9	55.8	0.990
PTV–LN_{mean}	68.3	68.5	0.708
V95–T (%)	99.6	99.8	0.114
V95–LN (%)	99.8	99.9	0.351

*All the dosimetric parameters of this subgroup were evaluated at primary planning, before the radiation boost.
